# Effectiveness of transcranial direct current stimulation on balance and gait in patients with multiple sclerosis: systematic review and meta-analysis of randomized clinical trials

**DOI:** 10.1186/s12984-023-01266-w

**Published:** 2023-10-24

**Authors:** Rafael Nombela-Cabrera, Soraya Pérez-Nombela, Juan Avendaño-Coy, Natalia Comino-Suárez, Rubén Arroyo-Fernández, Julio Gómez-Soriano, Diego Serrano-Muñoz

**Affiliations:** 1 Multiple Sclerosis Association of Torrijos, Torrijos, Spain; 2 Toledo Physiotherapy Research Group (GIFTO), Faculty of Physiotherapy and Nursing of Toledo, Universidad de Castilla-La Mancha, Toledo, Spain; 3 Physiotherapy Unit, Hospital Nuestra Señora del Prado, Talavera de la Reina, Spain; 4 Research Group on Water and Health (GIAS), Faculty of Physiotherapy and Nursing of Toledo, Universidad de Castilla-La Mancha, Toledo, Spain

**Keywords:** Multiple sclerosis, Transcranial direct-current stimulation, Gait, Balance, Rehabilitation

## Abstract

**Background:**

Motor impairments are very common in neurological diseases such as multiple sclerosis. Noninvasive brain stimulation could influence the motor function of patients.

**Objective:**

The aim of this meta-analysis was to evaluate the effectiveness of transcranial direct current stimulation (tDCS) on balance and gait ability in patients with multiple sclerosis. Additionally, a secondary aim was to compare the influence of the stimulation location of tDCS on current effectiveness.

**Methods:**

A search was conducted for randomized controlled trials published up to May 2023 comparing the application of tDCS versus a sham or control group. The primary outcome variables were balance and gait ability.

**Results:**

Eleven studies were included in the qualitative analysis, and ten were included in the quantitative analysis, which included 230 patients with multiple sclerosis. The average effect of tDCS on gait functionality was superior to that of the control group (SMD = -0.71; 95% CI, -1.05 to -0.37). However, the overall results of the tDCS vs. sham effect on static balance did not show significant differences between groups (MD = 1.26, 95% CI, -1.31 to 3.82). No significant differences were found when different locations of tDCS were compared.

**Conclusions:**

These results reveal that tDCS is an effective treatment for improving gait ability with a low quality of evidence. However, the application of tDCS has no effect on static balance in patients with multiple sclerosis with very low quality of evidence. Similarly, there seems to be no difference regarding the stimulation area with tDCS.

**Supplementary Information:**

The online version contains supplementary material available at 10.1186/s12984-023-01266-w.

## Introduction

Multiple sclerosis (MS) is a chronic, autoimmune, and inflammatory disease of the central nervous system characterized (CNS) by demyelination and axonal damage that originates in focal areas of injury, affecting both white and gray matter [1]. Neurological damage produces various symptoms, such as cognitive problems, sensory alterations, or pain, but the most frequent symptoms are postural instability, gait disturbances, spasticity, and fatigue [2, 3]. All these symptoms contribute to an increased risk of falls [4, 5], affecting daily activities and reducing activity and participation [6]. Therefore, recovering or improving balance, postural control, and gait will be essential in the management of patients with MS.

CNS impairments result in motor and sensory disturbances that lead to gait and balance impairments [7] All these alterations represent a reduction in postural control in patients with MS, resulting in postural instability that ultimately affects the gait pattern. Individuals with MS often exhibit a cautious gait pattern with the aim of reducing postural instability, but this attempt at postural control can increase the risk of falling [5, 8].

The approach to MS rehabilitation is recommended to be carried out from a multidisciplinary perspective, aiming to decrease physical and cognitive deterioration. Interventions include medication, exercise, and physical therapies [9, 10]. However, physical symptoms such as fatigue, muscle weakness or instability often do not respond to conventional interventions [11], and it is necessary to validate new therapeutic options to optimize standard rehabilitation [12].

Balance impairments can result from multiple causes, such as ataxia, weakness, spasticity, vision problems, proprioceptive deficits, and vestibular issues [2, 4]. Rehabilitation for balance and gait deficits follows the principles of neuroplasticity and motor learning strategies. The goal of rehabilitation is to minimize motor impairments while facilitating the activation of new neural pathways. Exercise is considered one of the most effective tools in traditional treatment for loss of balance [4, 13].

The emergence of non-invasive brain stimulation techniques, such as transcranial direct current stimulation (tDCS), has shown great potential in the field of neurorehabilitation [14]. By delivering low electrical currents to specific regions of the brain through scalp electrodes, tDCS can modulate neural activity and enhance the effects of various therapies [15].

Lefaucheur et al. summarized the effects of tDCS on the CNS. Anodal stimulation in the contralateral primary motor cortex (M1) increases output in the corticospinal tract, improving strength and increasing motor-evoked potential, which could explain the improvement in motor function of upper and lower limbs [16], being a recommended technique in the motor recovery process of neurological patients [17]. On the other hand, the cerebellum plays a prominent role in various brain functions, including postural control and gait. Mc Loughlin et al. showed that cerebellar dysfunction increases sway and instability in patients with MS, suggesting that interventions aimed at improving cerebellar function could result in better postural control for patients [18]. Finally, the cerebellum plays a crucial role in learning [19], in the planning and execution of movement by evaluating motor errors [20] and in the automation of movements through practice [21] as occurs in the case of walking.

This phenomenon opens up new possibilities for combining tDCS with existing treatments, such as motor recovery programs or cognitive rehabilitation. This technique has been used for the treatment of motor deficits to enhance the effects of traditional therapies [15].

Several modalities of tDCS application are distinguished. The most studied is anodal stimulation in the M1, although in recent years, anodal tDCS application in the cerebellum has also been investigated [14]. While some studies have found positive effects when combining tDCS with different physical therapies, other trials have shown contradictory results [14]. This inconsistency in the evidence may be related to the sample size of the studies, different assessment methods, or even the previous state of patients participating in the study. However, the exact influence of tDCS on gait and balance function in patients with MS remains unclear, as well as the most recommended parameters, dosage, and application area.

The main objective of this systematic review and meta-analysis was to summarize and synthesize the available evidence regarding the efficacy of tDCS for improving gait and balance function in patients with MS compared to sham. A secondary objective was to compare the influence of the location of tDCS on current effectiveness, specifically when applied to the primary motor cortex or the cerebellum.

## Methods

This systematic review and meta-analysis were conducted following the Preferred Reporting Items for Systematic Reviews and Meta-Analysis (PRISMA) and the recommendation by the Cochrane Collaboration. This protocol was registered in the PROSPERO database under the ID CRD42023424113.

### Search strategy

A search was conducted in the following databases until 31 May 2023: PubMed, the Physiotherapy Evidence Database (PEDro), the Cochrane Library, Scopus and Web of Science. The following keywords were used for the search: “transcranial direct current stimulation,“ “tDCS,“ “non-invasive brain stimulation,“ “multiple sclerosis,“ “balance,“ “gait,“ and “walking capacity.“ The terms “OR” and “AND” were used in combination with MeSH terms (see Additional file 1).

### Inclusion and exclusion criteria

The studies were selected based on the PICOS verification method (P-population; I-intervention; C-comparison; O-outcome and S-study design). Studies were included according to the following inclusion criteria: (1) randomized controlled trials (RCTs); (2) tDCS as the treatment; (3) intervention compared to sham tDCS; (4) includes at least one measure of balance or gait ability; and (5) the article was written in English or Spanish. The exclusion criteria were (1) preclinical trials and (2) studies where tDCS dosage and utilization were not specified.

After defining the search strategy, the studies were imported into reference management software (Mendeley, desktop version 2.75.0) to exclude duplicates. Two independent researchers (RNC and NCS) selected the studies based on the inclusion and exclusion criteria, with the involvement of a third researcher in case of disagreement (DSM).

### Data collection and extraction

A researcher (RNC) performed the screening and data extraction. Data from the studies were extracted in the following way: authors and year of publication, sample size, number of sessions and dose applied, stimulation location, assessment instrument, assessment time and type of MS.

### Risk of bias

The methodological quality of the RCTs included the recommendations from the Cochrane guidelines [22] using the Cochrane Risk of Bias tool (RoB 2.0), evaluating the risk of bias across five domains [23]: “randomization process”, “deviations from intended interventions”, “missing outcome data”, “measurement of the outcome”, and “selection of reported results.“ The risk of bias judgment for each of the five domains was assessed as “low risk of bias,“ “some concerns,“ or “high risk of bias.“ The “overall” risk was rated as “low risk of bias” if all domains were rated as low risk, “some concerns of bias” if at least one domain was rated with some concerns, and “high risk of bias” if at least one domain was rated as high risk. This questionnaire was used by two independent researchers (RNC and NCS). Any disagreements were resolved by a third researcher (DSM).

### Certainty of evidence. GRADE system

To assess the quality of evidence, the Grades of Recommendation Assessment, Development, and Evaluation (GRADE) system was used [24].

### Data synthesis and analysis

The inverse variance method was used for 4 variables: gait functionality, TUG, BBS, and MSWS-12. The statistical heterogeneity was assessed using the chi-square test (with statistical significance at p < 0.05) and calculated using I^2^, with 25%, 50%, and 75% representing low, moderate, and high heterogeneity, respectively.

A random-effects model was used when heterogeneity exceeded 50%, and a fixed-effects model was used when it was below 50%. The Mean Difference (MD) was used to express the outcome of the variables analyzed independently: TUG, BBS, and MSWS-12. These results were expressed in seconds for the TUG test and as a numerical score for the BBS and MSWS-12. A Standardized Mean Difference (SMD) was calculated with the changes in pre and posttreatment values from different studies and variables that assessed gait function. These results correspond to the assessment immediately after the last intervention conducted in each study. If the studies reported results with the mean postintervention, the mean change was calculated by subtracting the final mean from the baseline group mean. Standard deviations for the baseline change were calculated using a correlation coefficient (r) estimated at 0.7 as recommended by Cochrane, and the standard deviation of the baseline and final means for each group was calculated using the following equations [25]:

Mean change = final mean – baseline mean.

$$\text{S}\text{D} \text{C}\text{h}\text{a}\text{n}\text{g}\text{e} =\sqrt{\left(SD baseline\right)2+\left(SD final\right)2-\left(2 x r x SD baseline x SD final\right)}$$

The Risk Difference (RD) was calculated for adverse events and losses to follow-up. The confidence intervals were set at 95% (95% CI) for all variables. Intention-to-treat analysis was used. If studies involved more than two arms, only the analysis of the group receiving sham stimulation was included. The effect of the primary variable was analyzed by comparing tDCS with sham in the control group. An analysis was carried out including validated gait functionality variables in MS: the 2-minute walk test (2MWT) [26], the 25-foot walk test (25FWT) [27] and the Timed Up and Go test (TUG) [28]. When studies assessed multiple variables for gait functionality, TUG was prioritized as it is a validated scale in the MS population because it has shown a strong correlation with disability status (RS = 0.8) and a moderate correlation with balance and fall risk (RS = 0.66) [28]. Subsequently, a subgroup analysis was performed based on the stimulation location: M1 or cerebellum. Subsequently, an analysis by scales was conducted to observe if the heterogeneity from the first analysis decreased. Analysis by variables included the TUG, the Berg Balance Scale (BBS), and the 12-item Multiple Sclerosis Walking Scale (MSWS-12) [29]. At least two studies were required in each subgroup to establish a comparison. Review Manager (RevMan) software, version 5.4.1, Copenhagen, was used for the quantitative analysis. Evidence of publication bias was detected by visually inspecting funnel plot asymmetries.

## Results

### Study selection process

**Fig. 1:**
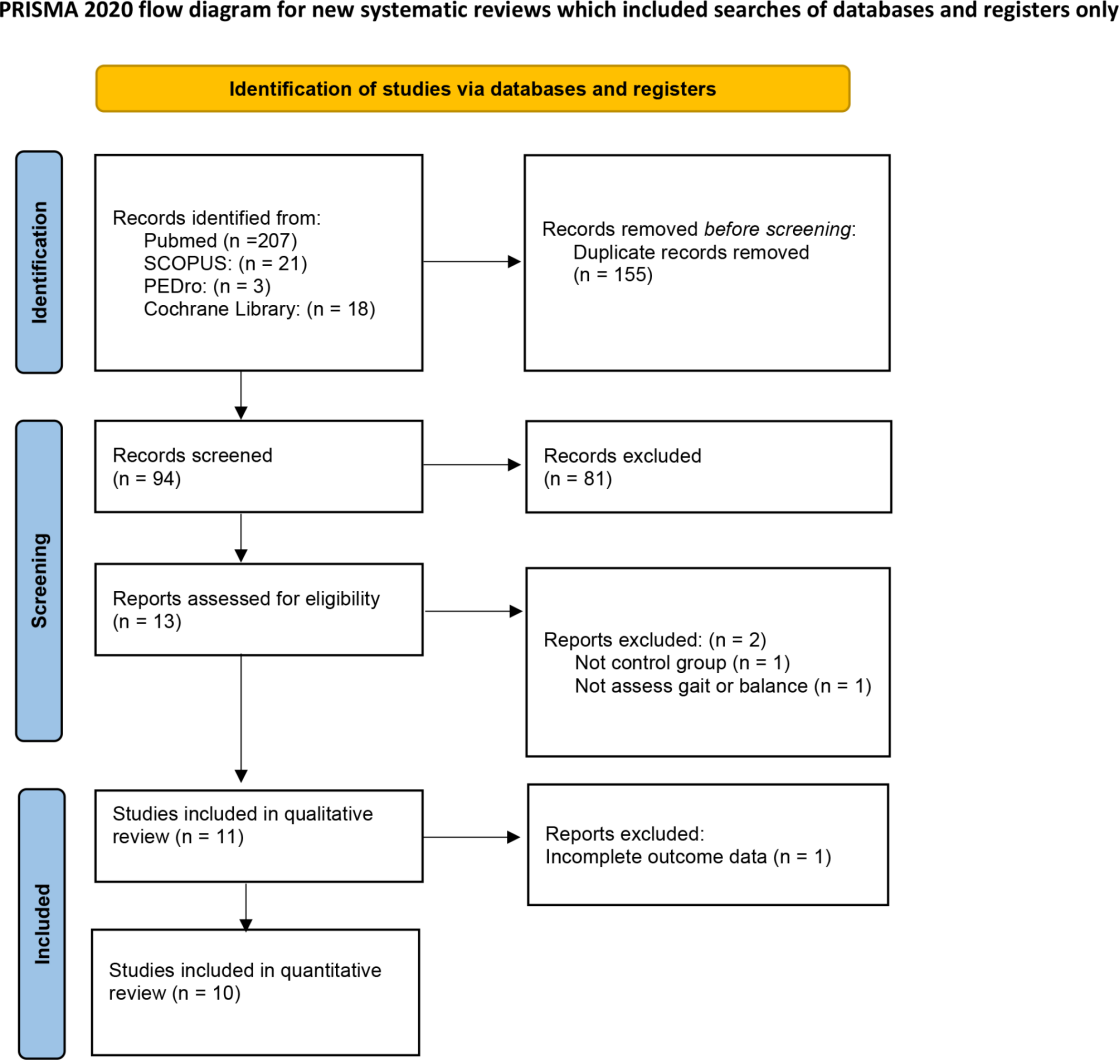
Flow chart of the study selection process

After removing duplicates, 94 clinical trials were identified as eligible, of which 81 were excluded after reading the title and abstract. Finally, after reading the full article, 11 RCTs were included [30–40] in this systematic review, and 10 RCTs were included in the meta-analysis. One of the articles [30] was excluded due to insufficient data available to include it in the quantitative analysis. (Fig. 1).

### Qualitative summary of included studies

The characteristics of the included studies are summarized in Table 1. All studies had a control or sham group. Of the 11 included studies, all had a parallel design, except for one that had a crossover design [30] (excluded from the quantitative analysis due to lack of data). The included studies combined the application of tDCS with other therapies, such as strength exercise, aerobic exercise, balance training, or abdominal belt exercises; treadmill training, task-oriented training, or stationary bike training; and conventional physiotherapy or postural training with the Byodex Balance system.

**Table 1:** Main characteristics and outcomes of the reviewed articles

Article	N (analysed)	N° sessions and tDCS parameters (duration, intensity, electrode area)	tDCS electrode placement	Variables	Design	Other therapy	MS type
Baroni et al., 2022	16 (16)	10 sessions 15 min 2 mA Electrodes of 35 cm^2^	+: cerebellum right hemisphere -: ipsilateral buccinator	TUG 8-feet test DGI MSWS-12	Pre Post After 2 weeks	Task-oriented training	RR PP SP
Ehsani et al., 2022	37 (30)	10 sessions 20 min 1.5 mA Electrodes of 35 cm^2^	+: cerebellum right hemisphere -: right buccinator	Biodex balance system Berg Scale FES-1	Pre Post After 1 month	Postural training	
Iodice et al., 2015	20 (20)	5 sessions 20 min 2 mA Electrodes of 35 cm^2^	+: M1 contralateral to the most affected side -: supraorbital area contralateral to the anode	MSWS-12	Pre Post After 1 week		RR
Marotta et al., 2022	17 (17)	10 sessions 20 min 2.5 mA Electrodes of 25 cm^2^	+: M1 (C3) -: supraorbital area	TUG 6MWT Berg Scale FRI	Pre Post After 4 weeks After 6 weeks	Conventional physical therapy	RR
Mohammadkhabeigi et al., 2022	29 (29)	5 sessions 20 min 2 mA Electrodes of 25cm^2^	+: M1 (CZ) -: supraorbital area	Berg Scale TUG 6MWT	Pre Post	Core stability training	RR
Nguemeni et al., 2022	22 (22)	6 sessions for 2 weeks 15 min 2 mA Electrodes of 35 cm^2^	+: cerebellum ipsilateral to the most affected -: ipsilateral buccinator	FGA TUG 2-min walking test 50-meters walking test	Pre Post After 6 weeks	Split Belt Treadmill	
Oveisgharan et al., 2019	17 (13)	7 sessions 30 min 2.5 mA/cm^2^ Electrodes of 16 cm^2^	+: M1 (CZ) -: inion	T25FW MSWS-12	Pre Post		RR
Pilloni et al., 2020 (1)	17 (17)	1 session 20 min 2.5 mA/cm^2^ Electrodes of 25 cm^2^	+: M1 (C3) -: contralateral supraorbital area	10MWT TUG	Pre Post	Aerobic exercise	RR PP
Pilloni et al., 2020 (2)	18 (15)	10 sessions 20 min 2.5 mA/cm^2^ Electrodes of 25 cm^2^	+: M1 (C3) -: FP2	10MWT 2-min walking test MSWS-12	Pre Post	Aerobic exercise	RR PP
Rahimibarghani et al., 2022	50 (39)	12 sessions 25 min 1.5 mA Electrodes of 16 cm^2^	+: M1 dominant hemisphere (C3) -: contralateral shoulder	TUG 2-min walking test 5-min walking test	Pre Post After 1 month	Exercise on a stationary bike	RR PP SP
Workman et al., 2019	12 (12)	1 session 13 min 2 mA/cm^2^ Electrodes of 25 cm^2^	+: contralateral M1 to the most leg affected (C3-C4) -: contralateral supraorbital area	6MWT	Pre Post		RR

6MWT: 6-minute Walking Test; 10MWT: 10-minute Walking Test; DGI: Dynamic Gait Index; FGA: Functional Gait Assessment; FP: Frontal Polar; M1: Primary Motor Cortex; MSWS-12: Multiple Sclerosis Walking Scale 12-items; PP: Primary Progressive; RR: Remittent Recurrent; SP: Secondary Progressive; T25FW: 25-feet Walking Test; TUG: Timed Up and Go.

This systematic review included 11 RCTs with a total sample of 230 participants for qualitative analysis, of whom 156 (67.82%) were females, with a mean age of 40.3 years for the entire sample. The types of MS included in the studies were as follows: relapsing-remitting MS in 9 studies [30–33, 35–38, 40], primary progressive MS in 4 studies [30, 32, 33, 36], and secondary progressive MS in 2 studies [36, 38]. None of studies included recurrent progressive MS. Out of the 11 included studies, eight applied the McDonald criteria for MS diagnosis and participant inclusion in the study [41]. The remaining studies did not specify the criteria they used for diagnosing MS.

All included studies, except the one by Workman et al. [30], used the Expanded Disability Status Scale (EDSS) [42] to assess the functional status of the study subjects. The results of the scale range from 1 to 6.5, indicating that all included participants were able to walk with assistance. The study by Workman et al. [30] used the Patient-Determined Disease Scale (PDDS), and the results for the patients ranged from 2 to 6 points, corresponding to moderate disability.

Regarding the stimulation protocol, all studies used anodal tDCS. Eight studies [30–33, 35–37, 40] applied stimulation over M1, with the cathode placed over the supraorbital region or the shoulder region. The remaining three studies applied stimulation over the cerebellum, with the cathode placed on the buccinator muscle [34, 38, 39]. Five of the studies used electrode sizes of 25 cm^2^ [30, 32, 33, 35, 37], four studies [34, 38–40] used 35 cm^2^ electrodes, and only two studies [31, 36] used 16 cm^2^ electrodes. The current intensity varied between 1.5 mA and 2.5 mA. The applied current density ranged from 0.04 mA/cm^2^ [34], 0.05 mA/cm^2^ [38, 40], 0.08 mA/cm^2^ [30, 35, 37, 39], 0.09 mA/cm^2^ [36], 0.1 mA/cm^2^ [32, 33], to 1.5 mA/cm^2^ [31], which was the highest current intensity used.

The duration of tDCS stimulation sessions varied between 13 and 30 min. The 20-minute application time was the most used by the authors [32–35, 37, 40]. Workman et al. used the shortest stimulation time of 13 min [30]. Two articles applied 15 min of stimulation [38, 39]. One study applied stimulation for 25 min [36], and Oveisgharan et al. used the longest stimulation time of 30 min [31]. The frequency of sessions varied from two to seven per week, with a frequency of 5 sessions per week being the most common [32–34, 37, 38, 40]. The total number of sessions ranged from a single session to 12 sessions. The follow-up period was equal to or less than one and a half months in all studies, with no long-term follow-up in 5 of the studies [30–33, 37].

Regarding the blinding protocol, all articles implemented similar setups between the real stimulation and sham groups, also keeping the sham stimulation time equal to the real stimulation time. Six articles used a 30-second ramp-up at the beginning of sham stimulation [30, 31, 34, 36, 38, 40] and three articles used a 60-second ramp-up [32, 33, 37] to simulate the initial itching that is felt with tDCS and then disconnecting the current. This type of protocol has been described as effective for conducting double-blind clinical trials in neurorehabilitation [43]. Only two articles did not specify whether they used the initial ramp-up before disconnecting the current in the sham group [35, 39].

The functional assessment of gait was the main variable measured in eight studies. In 6 studies, it was measured using the TUG test [32, 33, 35–39]. Two articles used validated gait assessment scales such as the 25FWT [31] and the 2MWT [33]. The secondary variables included were the balance assessment using the BBS [34, 35, 37] and perceived gait assessment using the MSWS-12 [33, 38, 40]. Other included variables were not within the protocol objectives for this systematic review and meta-analysis. The number of patients reported as lost to follow-up was 21 (6.8%). No serious adverse effects were reported during tDCS.

### Risk of bias

**Fig. 2:**
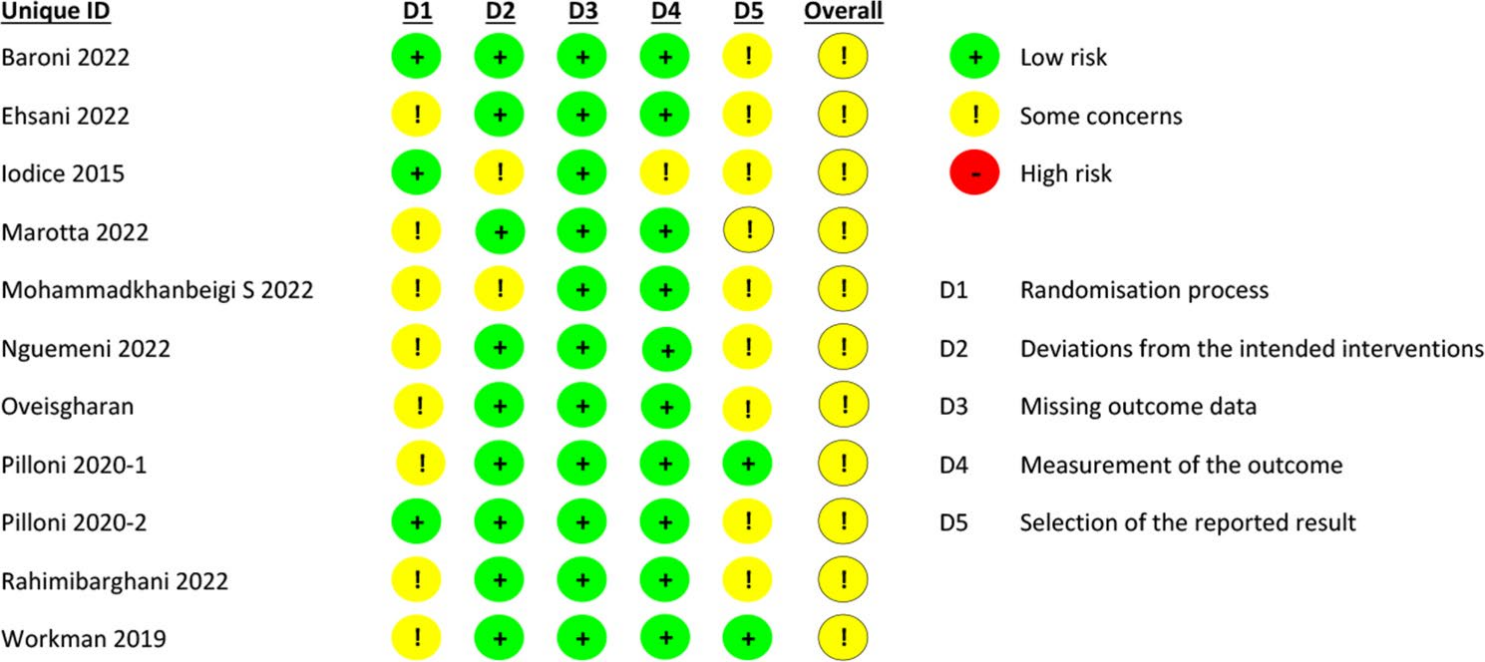
Analysis of the risk of bias according to the authors’ judgments on each item assessing the risk of bias for each included study

Figure 2 shows the risk of bias of the studies included in the systematic review and meta-analysis. The two investigators who assessed the risk of bias (RNC and NCS) agreed on 82% of the items. In general, all included studies had some problems with risk of bias. The results of Egger’s test were significant for balance and gait functionality, suggesting a possible risk of publication bias in the comparison of tDCS and sham. (see Additional file 2).

### Quantitative summary: effects of transcranial direct current stimulation

#### Effects on functional gait assessment

**Fig. 3:**
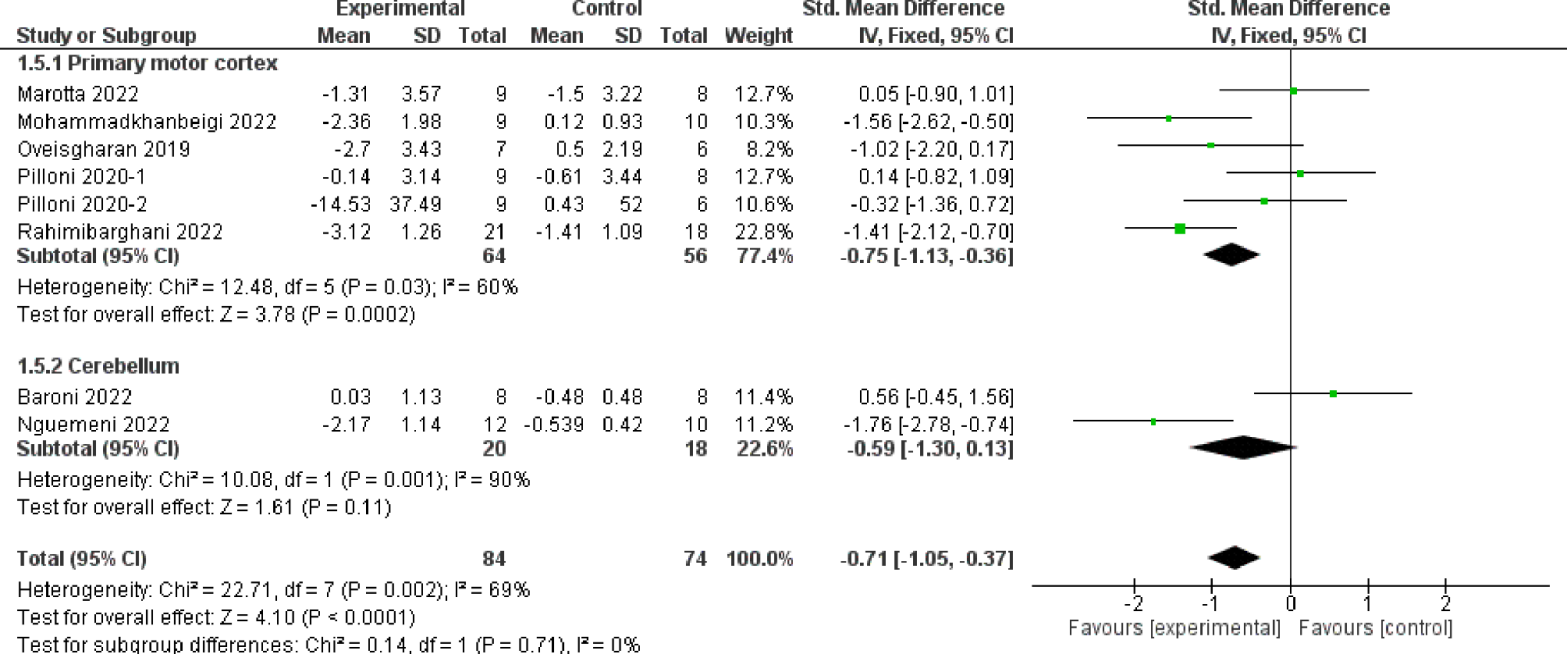
Result of meta-analysis for effect of tDCS versus sham on gait functionality

The meta-analysis of 8 articles and 158 subjects is summarized in Fig. 3 and includes trials that assessed the effect of intervention on various functional gait variables: TUG, 2MWT, and 25FWT. The average effect of tDCS on functional gait was superior to the control group (SMD = -0.71; 95% CI, -1.05 to -0.37), with a high level of heterogeneity (I^2^ = 69%, p = 0.002). The values for M1 stimulation were (SMD = -0.75; 95% CI, -1.13 to -0.36), with a high level of heterogeneity (I^2^ = 60%, p = 0.03). No statistically significant differences were found in the cerebellum stimulation (SMD = -0.59; 95% CI, -1.3 to 0.13), with very high heterogeneity (I^2^ = 90%, p = 0.001). Additionally, the subgroup analysis comparing the stimulation site did not observe significant differences between the M1 and cerebellum (p = 0.76). Overall, evaluating all the articles, the effect size, according to Cohen’s d, was − 0.71, which indicates a moderate effect [44]. The quality of evidence for this outcome according to GRADE was low in terms of factors to rate down (serious inconsistency or heterogeneity and Egger’s test significant).

#### Effects on timed up and go

**Fig. 4:**
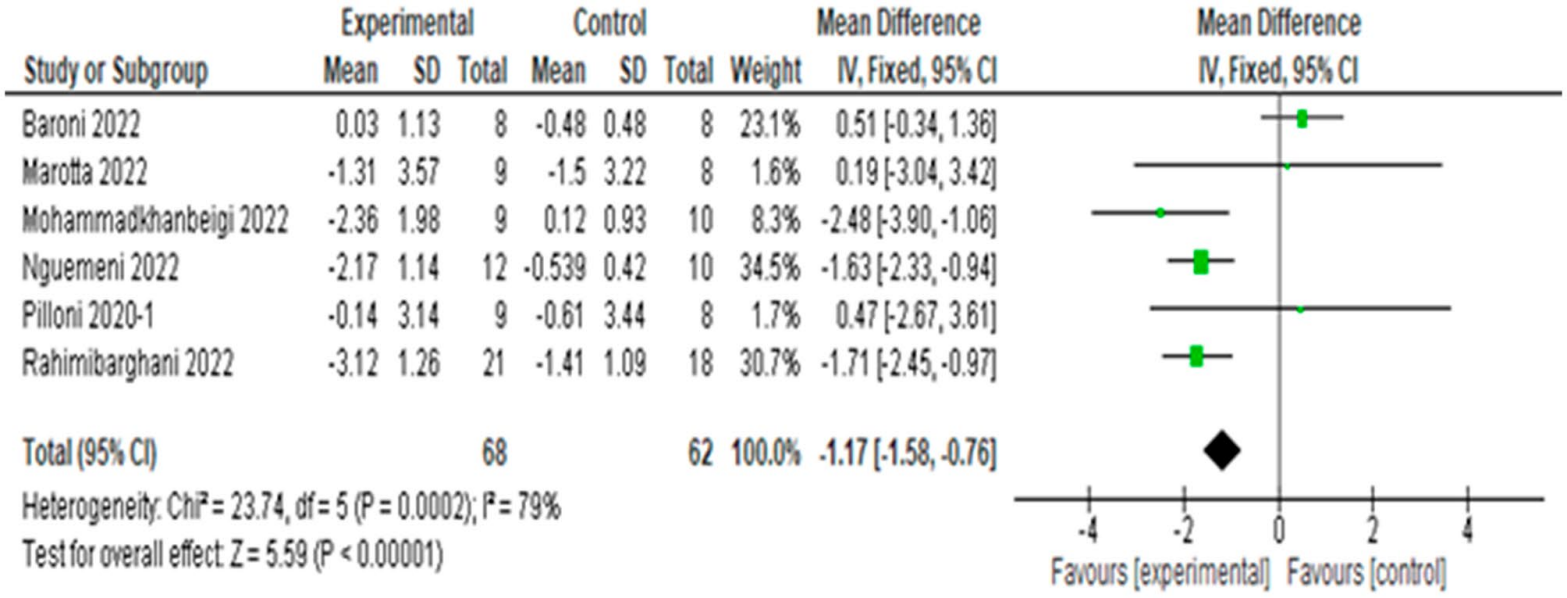
Result of meta-analysis for effect of tDCS versus sham on the TUG test

The meta-analysis of 6 articles and 130 subjects is shown in Fig. 4. To reduce heterogeneity, an analysis by scale was performed. The overall results of the effect of tDCS vs. sham tDCS on the TUG were favorable to the real stimulation group (MD = -1.17 s., 95% CI, -1.58 to -0.76), with a high level of heterogeneity (I^2^ = 79%, p < 0.001). The quality of evidence for this outcome according to GRADE was low in terms of factors to rate down (serious inconsistency or heterogeneity and Egger’s test significant).

#### Effects on the BBS

**Fig. 5:**
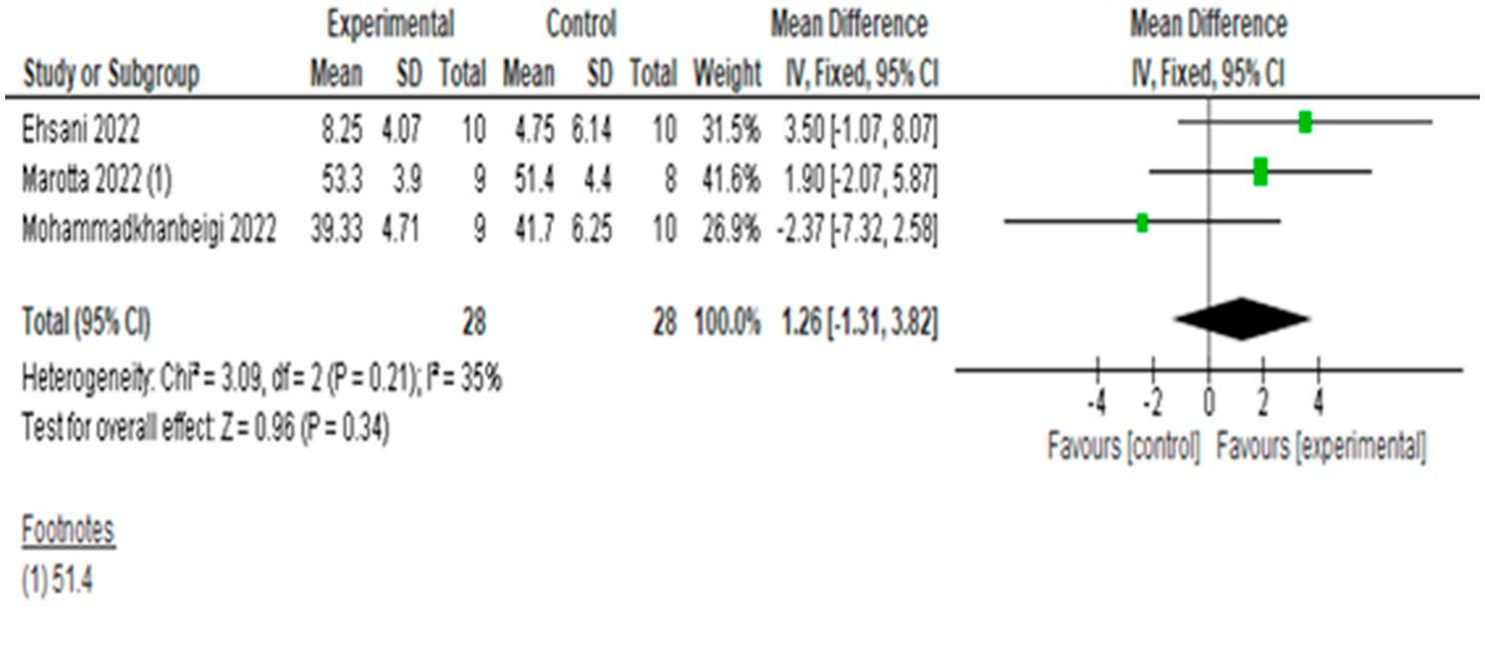
Result of meta-analysis for effect of tDCS versus sham on the BBS.

The meta-analysis of 3 articles and 56 subjects is shown in Fig. 5. The overall results of the effect of tDCS versus sham tDCS on the BBS showed no difference between the groups (MD = 1.26 points, 95% CI, -1.31 to 3.82), with a very low level of heterogeneity (I^2^ = 35%, p = 0.21). The quality of evidence for this outcome according to GRADE was low in terms of factors to rate down (very serious imprecision because the sample was very small and the confidence intervals wide).

#### Effects on the MSWS-12

**Fig. 6:**
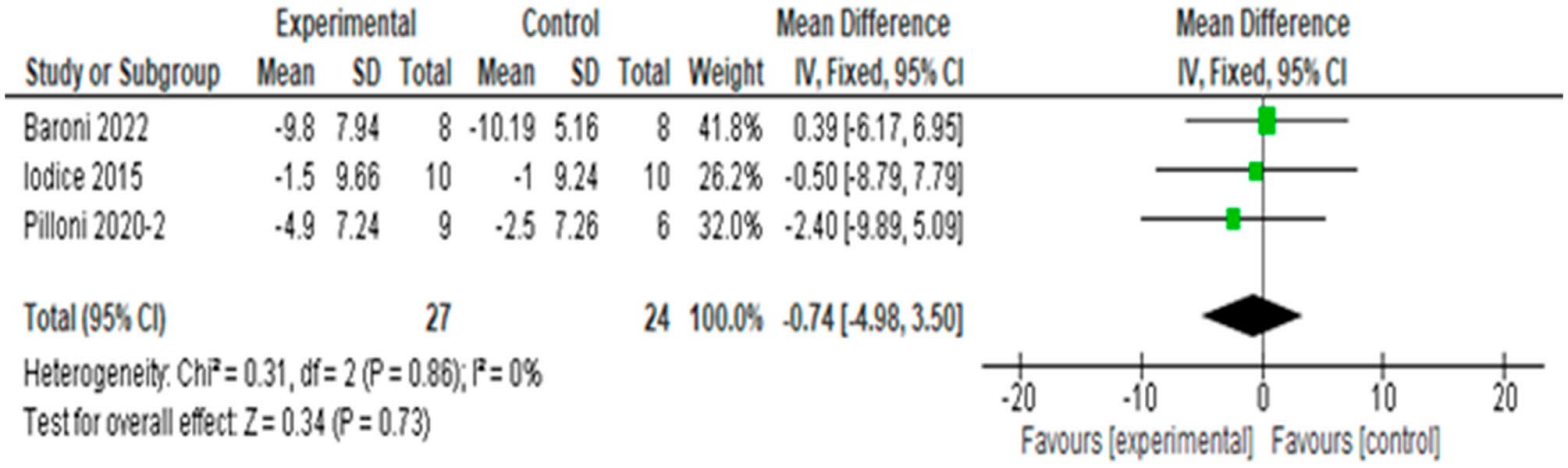
Result of meta-analysis for effect of tDCS versus sham on MSWS

The meta-analysis of 3 articles and 61 subjects is shown in Fig. 6. The overall results of the effect of tDCS versus sham tDCS on the MSWS-12 scale found no differences between the groups (MD = 0.09 points, 95% CI, -4.73 to 4.91), with no heterogeneity (I^2^ = 0%, p = 0.86). The quality of evidence for this outcome according to GRADE was low in terms of factors to rate down (very serious imprecision because the sample was very small and the confidence intervals wide).

## Discussion

This systematic review and meta-analysis included 11 clinical trials with a total sample of 230 participants to assess the effect of tDCS on gait and balance in patients with MS. Most of the patients included in the meta-analysis had a course of relapsing-remitting MS, although the type of MS does not appear to be a determining factor [45]. The results of the meta-analysis showed a general improvement in the scores of the set of gait functionality variables (TUG, 25FWT, 2MWT). Regarding the static balance variable, assessed with the BBS, no significant differences were found. Similarly, no significant differences were found in the self-perception of gait variable (MSWS-12). Furthermore, no significant differences were found when different locations of current application of tDCS were compared.

To date, there has been only one systematic review and meta-analysis evaluating gait and balance function in MS patients after cerebral stimulation [46], and no significant differences were found. However, in the present study, tDCS therapy produced statistically significant improvements in gait function compared to sham stimulation. The absence of differences in the review by Emadi et al. [46] may be due to the combination of different forms of non-invasive cerebral stimulation, tDCS and transcranial magnetic stimulation. In the current literature, the existing evidence is contradictory regarding the effect of tDCS on gait and balance, as multiple systematic reviews on the use of tDCS in MS focus on other symptoms. Regarding lower limb motor function, Kan et al. [47] included only 4 RCTs that used tDCS, but they did not conduct a meta-analysis due to the lack of data from the included studies.

The possible mechanisms of action of non-invasive brain stimulation with tDCS in MS are related to neuronal plasticity processes. The focal lesions that occur in MS, known as plaques, which have been evidenced in studies with magnetic resonance imaging [3], may lead to alterations in cortical excitability causing certain symptoms of MS, making the regulation of cortical excitability a therapeutic approach. tDCS by increasing the cortical excitability of M1, can generate greater activity of neural circuits that induce neuroplastic processes in the networks involved in human gait, as well as the different control centers of the CNS, such as the cerebral cortex, cerebellum or basal ganglia [16].

For these neuroplastic effects in the brain to persist beyond the stimulation time, tDCS should be sustained (minutes), as it induces changes that endure for hours [16]. Brief stimulation (seconds) produces these changes, but they do not persist over time. It seems that this persistence may be involved in the modification of synaptic activity and the neuroplastic mechanisms of long-term potentiation or depression of synaptic transmission in neuronal networks [48].

Studying the application of tDCS using magnetic resonance imaging [49], it has been observed that after stimulation, the connectivity between different brain areas is increased, involving networks that were previously in a resting state. tDCS reconfigures brain networks, modulating cortical excitability, which influences the activity of neuronal networks, making it highly valuable for neurorehabilitation. Neural networks respond to electric fields, so tDCS could affect functional connectivity and activity at various levels, both cortical and subcortical, involved in the neurophysiological control of gait [50].

Additionally, tDCS can influence various pathological processes that occur in the central nervous system [16]. On the one hand, nonsynaptic mechanisms in the nervous system may be based on changes in protein channel density or ion conductance, such as K + or Ca2+. It can also act on axon molecules or neurotransmitters due to its polarity [16, 51].

On the other hand, nonneural tissues also react to the application of electrical current; for instance, the anode can lead to vasodilation of cerebral capillaries [52]. Additionally, cells such as lymphocytes or glial cells have their activity modified by tDCS [53]. Among the functions of glial cells is the myelination of axons, this could be an interesting point in the application for patients with MS, as, at least in theory, brain stimulation may influence demyelination and improve nerve transmission, which could explain its effect on gait. Finally, it has been observed that stimulation can activate other neuroplastic mechanisms such as axonal regeneration and neuronal growth [54].

More recently, a type of cerebellar cortex stimulation has emerged [16]. Although in our study, no significant differences were observed compared to sham stimulation in gait functionality when tDCS was applied at the cerebellar level, this approach could be a preferred intervention in different pathologies.

There are theoretical models in the literature that support the idea that the electric field generated by tDCS can reach the cerebellum using an appropriate montage [55]. However, various factors can influence the distribution of the tDCS. Workman et al. [56] showed in a PET study, that the cortical response to tDCS might be site-specific and may require different stimulation parameters (e.g., current intensity, electrode orientation) to appropriately excite/inhibit the different target regions. Furthermore, DLPFC and M1 might have different cortical orientations/alignments or neuronal compositions/morphologies that could also contribute to site-specificity.

Cerebellar stimulation may influence gait adaptation [57] and motor learning [58] in healthy subjects, recommending its use in rehabilitation treatment. However, only one study has measured the efficacy of cerebellar tDCS to improve motor learning consolidation in patients with MS, without finding statistically significant differences between real and sham stimulation [59], which aligns with the findings of this study. Therefore, future trials will be necessary to study the feasibility of this approach.

The goal of individualizing treatment should take priority in tDCS therapy, as numerous characteristics influence cerebral plasticity, such as exercise, age, attention capacity, sex, or medications [60]. Several studies have shown different responses to tDCS between sexes for cognitive [61] or motor aspects [62]. These differences could be explained by hormone levels, anatomical variations of the cranium, or differences in cortical excitability [63]. However, it is difficult to make any assumptions regarding sex in this study because the sample was very balanced (68% women), and the analyzed studies did not differentiate between sexes. Regarding age, it also influences the plastic changes induced by tDCS, with elderly subjects responding less favorably to tDCS [60, 64]. However, the mean age of our included sample is middle-aged (40.3 years old), so it may not be a limiting factor for the meta-analysis results.

Nevertheless, it is necessary to emphasize that there is a wide variability in the response to tDCS stimulation, which can influence the clinical outcomes obtained. Additionally, the clinical changes observed by tDCS may be strongly influenced by individual neuroanatomical variations. All these factors could explain the variable results provided by trials in pathological conditions and the difficult optimization of tDCS parameters.

A possible useful tool for individualizing treatment could be electroencephalography (EEG), as it measures brain activation [65]. It has been observed that patients with MS had lower frequency and amplitude values in the EEG than controls, there are neurological pathologies that are associated with the alteration of these brain rhythms, so their physiological restoration could be clinically relevant for the gait function [66]. EEG detects the voltage of neuronal activity and could be applied simultaneously with electrical stimulation, allowing for dose adjustment (current density and duration) so that each patient reaches their optimal cortical excitability level and, in theory, detect patients in whom tDCS causes a null response or response opposite to what was expected. However, none of the studies included in the present review have used this type of tools to individualize the stimulation parameters probably due to the complexity of recording and interpreting this signal.

Future tDCS studies should aim to identify patients who are more likely to benefit from stimulation. As already seen, there are several ways to quantify cortical changes: neurophysiological studies of TMS-induced motor evoked potential, through neuroimaging studies such as functional magnetic resonance imaging or positron emission tomography (for spatial assessment), or EEG (for temporal assessment) [67].

Regarding the clinical implications, based on the data found in our meta-analysis, the moderate quality of evidence suggests that tDCS versus control may improve gait function but not static balance. One possible reason for not finding significant improvements in static balance may be the use of the BBS, as it can have a ceiling effect in patients who are not severely affected, as occurs in patients with Parkinson’s disease [68]. The effect size found was moderate (0.71) according to Cohen’s d for functionality, with an observed improvement of 1.17 s in the TUG. The values of the minimal detectable change (MDC) for TUG in MS were calculated at 10.6 s [69]. This study recognizes that the variability of the patients and their low statistical power may be giving too broad a result and recommends future investigations to determine what improvement in TUG should be considered clinically significant. When compared with other neurological conditions, the variation is considerable; for example, in stroke patients, it is 2.9 s [70], and in Parkinson’s disease, it is 3.5 s [71], values closer to those found in this study.

The minimal clinically important difference (MCID) has not been studied in the population with MS for the TUG variable. The MCID is a method that defines the smallest difference in an outcome that patients and clinicians perceive as beneficial and not trivial. It is one of the necessary measures to interpret changes after therapeutic interventions. However, we can compare it with the MCID for TUG in the elderly population with hip osteoarthritis, which was established between − 0.8 and − 1.4 s [72]. Based on the data from our meta-analysis and always taking caution with the established comparisons, we can say that tDCS has clinically significant improvements, as it obtained an improvement of 1.17 s.

This study has some limitations that must be acknowledged. First, the quality of this evidence was downgraded due to the high levels of heterogeneity among the studies, making the comparability of studies difficult. Second, studies were included if they were written in English or Spanish. Languages in which there was also scientific literature on this area were not included and could be relevant for this meta-analysis and could interfere with the final result. Third, although all studies included sham stimulation to blind subjects, none of the studies performed an adequate assessment of the success of blinding. Studies with intensities higher than 1mA could be more difficult to blind [73] It is recommended to analyze the efficacy of blinding using specific methods such as Bang’s and James’s index [74, 75]. Finally, the potential publication bias observed in the Egger’s test should also be taken into account in order to interpret the results with caution. .

## Conclusion

According to the results of our meta-analysis, tDCS improves functional gait capacity in patients with MS with a low quality of evidence but not static balance with a very low quality of evidence. Similarly, there were no significant differences between stimulation in the M1 or cerebellum. Further studies with parameter standardization and individualization of application are needed to increase the success of tDCS therapy.

### Electronic supplementary material

Below is the link to the electronic supplementary material.

Supplementary Material 1

Supplementary Material 2

## Data Availability

The data collected in this study are available from the corresponding author on reasonable request. All primary data were extracted from the referenced sources.

## Abbreviations

BBS: Berg Balance Scale
CNS: Central nervous system
EDSS: Expanded Disability Status Scale
EEG: Electroencephalography
M1: Primary motor cortex
MD: Mean Difference
MDC: Minimal detectable change
MCID: Minimal Clinically Important Difference
MS: Multiple sclerosis
MSWS-12: 12-item Multiple Sclerosis Walking Scale
PDDS: Patient-Determined Disease Scale, PEDro:Physiotherapy Evidence Database
RCTs: Randomized controlled trials
RD: Risk Difference
SMD: Standardized Mean Difference
tDCS: Transcranial direct current stimulation
TUG: Timed Up and Go test
2MWT: 2-minute walk test
25FWT: 25-foot walk test
