# Supplementary material for: Effectiveness of transcranial direct current stimulation on balance and gait in patients with multiple sclerosis: systematic review and meta-analysis of randomized clinical trials
Source: J Neuroeng Rehabil. 2023 Oct 24;20:142. doi: 10.1186/s12984-023-01266-w (PMC10594930; doi:10.1186/s12984-023-01266-w)
Supplement: Supplementary file 1 — Supplementary Material 1 [file 12984_2023_1266_MOESM1_ESM.docx]

**Additional file 1. Search strategy.**

**PUBMED**

Keywords: transcranial direct current stimulation, tDCS, non-invasive brain stimulation, multiple sclerosis, balance, gait, walking capacity. The terms "OR" and "AND" were used in combination with the MeSH terms.

**Advance Search**

- (((((("transcranial direct current stimulation"[Title/Abstract]) OR ("tDCS"[Title/Abstract])) OR ("non-invasive brain stimulation"[Title/Abstract])) AND ("multiple sclerosis"[Title/Abstract])) AND (balance[Title/Abstract])) OR ("gait"[Title/Abstract])) OR ("walking capacity"[Title/Abstract])= 65098 results.
- (((((("transcranial direct current stimulation"[Title/Abstract]) OR ("tDCS"[Title/Abstract])) OR ("non-invasive brain stimulation"[Title/Abstract])) AND ("multiple sclerosis"[Title/Abstract])) AND (balance[Title/Abstract])) OR ("gait"[Title/Abstract])) OR ("walking capacity"[Title/Abstract]). Filtro “Randomized clinical trials” = 3,421 results
- (((((("transcranial direct current stimulation"[Title/Abstract]) OR ("tDCS"[Title/Abstract])) OR ("non-invasive brain stimulation"[Title/Abstract])) AND ("multiple sclerosis"[MeSH Major Topic]))) AND (gait[Title/Abstract])) OR ("walking capacity"[Title/Abstract]) Filtro “Randomized clinical trials”🡪 207 results

**PEDro**

- “tDCS” AND “multiple sclerosis”🡪 3
- "Transcranial direct current stimulation" AND "multiple sclerosis"🡪3
- ("tdcs") AND ("transcranial direct current stimulation") AND ("multiple sclerosis")🡪3

**COCHRANE LIBRARY**

- (("tDCS") OR ("transcranial direct current stimulation") AND ("multiple sclerosis") OR ("sclerosis") AND ("Gait") OR ("balance")):ti,ab,kw 🡪18527
- tdcs and multiple sclerosis and gait: ti,ab,kw🡪 19
- tdcs and multiple sclerosis and balance: ti,ab,kw 🡪 18

**SCOPUS**

- TITLE-ABS-KEY ( ( "tdcs" ) OR ( "transcranial direct current stimulation" ) AND ( "multiple sclerosis" ) OR ( "sclerosis" ) AND ( "Gait" ) OR ( "balance" ) )🡪 27
- TITLE-ABS-KEY ( ( "transcranial direct current stimulation" ) AND ( "multiple sclerosis" ) OR ( "sclerosis" ) AND ( "Gait" )🡪18
- TITLE-ABS-KEY (( "transcranial direct current stimulation" ) AND ( "multiple sclerosis" ) OR ( "sclerosis" ) AND ( "BALANCE" )🡪21

**WEB OF SCIENCE:**

- ((TI=((tdcs) OR (transcranial direct current stimulation))) AND TI=(("multiple sclerosis"))) AND TI=((gait) OR (balance))🡪8
