# Supplementary figures and images for: Effectiveness of transcranial direct current stimulation on balance and gait in patients with multiple sclerosis: systematic review and meta-analysis of randomized clinical trials
Source: J Neuroeng Rehabil. 2023 Oct 24;20:142. doi: 10.1186/s12984-023-01266-w (PMC10594930; doi:10.1186/s12984-023-01266-w)

**Additional file 2**. Egger test. Funnel plot publication risk of bias.


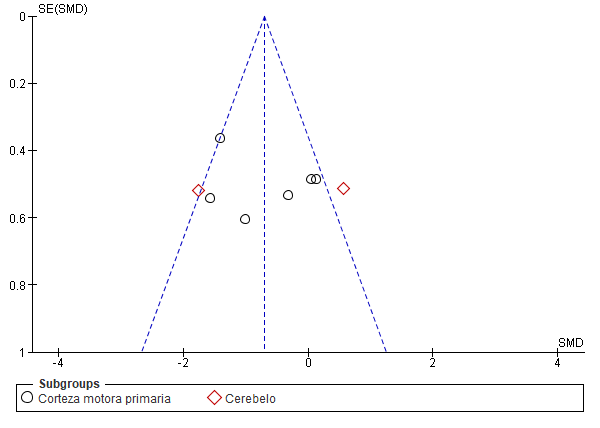

Supplement: Supplementary file 2 — Supplementary Material 2 [file 12984_2023_1266_MOESM2_ESM.docx]
